# Analysis of force-deconvolution methods in frequency-modulation atomic force microscopy

**DOI:** 10.3762/bjnano.3.27

**Published:** 2012-03-14

**Authors:** Joachim Welker, Esther Illek, Franz J Giessibl

**Affiliations:** 1 Institute of Experimental and Applied Physics, Experimental Nanoscience, University of Regensburg, Universitaetsstrasse 31, 93053 Regensburg, Germany

**Keywords:** frequency-modulation atomic force microscopy, force deconvolution, numerical implementation

## Abstract

In frequency-modulation atomic force microscopy the direct observable is the frequency shift of an oscillating cantilever in a force field. This frequency shift is not a direct measure of the actual force, and thus, to obtain the force, deconvolution methods are necessary. Two prominent methods proposed by Sader and Jarvis (Sader–Jarvis method) and Giessibl (matrix method) are investigated with respect to the deconvolution quality. Both methods show a nontrivial dependence of the deconvolution quality on the oscillation amplitude. The matrix method exhibits spikelike features originating from a numerical artifact. By interpolation of the data, the spikelike features can be circumvented. The Sader–Jarvis method has a continuous amplitude dependence showing two minima and one maximum, which is an inherent property of the deconvolution algorithm. The optimal deconvolution depends on the ratio of the amplitude and the characteristic decay length of the force for the Sader–Jarvis method. However, the matrix method generally provides the higher deconvolution quality.

## Introduction

The atomic force microscope (AFM) was invented 25 years ago as an offspring of the scanning tunneling microscope (STM), extending the imaging capabilities to insulators [1]. Nowadays the focus of development and investigation shifts from purely topographic imaging, in spite of this still being the main use of an AFM, to quantitative force measurements between single atoms or molecules in high-resolution, dynamic AFM modes. Examples are the measurement of the force needed to move an atom on surface [2] or the chemical identification of different adatom species [3]. Another trend is the three-dimensional force mapping [4–5] giving tomographic insight into the force field over atoms and molecules. However, all these remarkable results have to rely on inversion methods as the force is not directly measured in the dynamic modes of an AFM.

For high-resolution atomic force microscopy commonly the frequency-modulation (FM) technique is used [6]. In FM-AFM the direct observable is the frequency change of an oscillating cantilever due to the force field acting between the tip of the probe and the sample surface. The corresponding frequency shift is related to the actual force by a convolution [7]. Hence to obtain the force, deconvolution methods are necessary.

A number of inversion methods from frequency shift to force have been suggested. Iterative methods were proposed by Gotsmann [8] and Dürig [9]. The higher harmonics of the cantilever oscillation can be exploited to recover the force instantaneously [10]. Hölscher showed that a deconvolution is possible if the amplitude dependence of the frequency shift is known [11]. Predominantly, the direct deconvolution methods of the Δ*f*(*z*) dependency that were proposed by Sader and Jarvis [12] and Giessibl [13] are used. These methods were found to be the most robust [14]. Both methods start from the same equation for the convolution, but they have different approaches in solving it for the force.

In this paper we compare the Sader–Jarvis deconvolution method and Giessibl’s matrix method. We use the analytical formulas of the Morse and Lennard-Jones model forces and the corresponding frequency shifts. The analytically calculated frequency shifts are deconvoluted back into a force and compared with the original model force.

In the first section we introduce the model forces and the corresponding frequency-shift curves. In the second section both deconvolution methods and their implementation for discrete data points are described. In the third section we present the results of the simulation showing a nontrivial amplitude dependence of the deconvolution quality and discuss the origin of the variations in deconvolution quality.

### Forces and frequency shifts in FM-AFM

In FM-AFM the force is not directly proportional to the measured frequency shift, but instead to the average force gradient, as can be seen from a simple model. Let us assume an interaction potential between a tip and a sample denoted by *V_ts_*(*z*). Accordingly, the force is given by *F_ts_*(*z*) = −(d*V_ts_*(*z*)/d*z*) and the force gradient by *k_ts_*(*z*) = −(d*F_ts_*(*z*)/d*z*). If *k_ts_* is constant over the range of one oscillation cycle, which is fulfilled, for example, for small amplitudes, the actual resonance frequency *f* can be calculated with an effective spring constant *k* + *k_ts_*

[1]
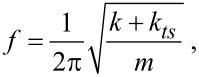


where *m* is the effective mass and *k* the spring constant of the cantilever. For *k_ts_* << *k* we can expand the square root in Equation 1 and calculate the frequency shift Δ*f* = *f* − *f*_0_

[2]
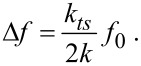


**Figure 1:**
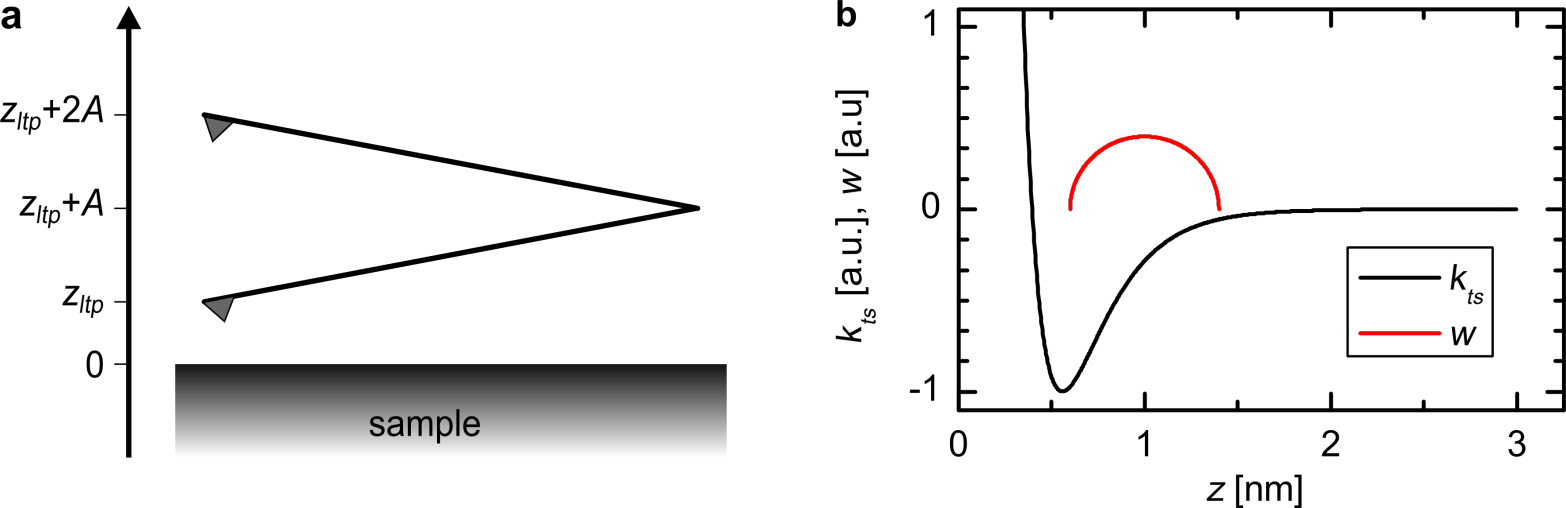
(a) Definition of the *z*-axis: The cantilever oscillates with a constant amplitude *A*. The lower turnaround point is denoted with *z_ltp_* and the center of the oscillation is at *z_ltp_* + *A*. (b) The frequency shift can be calculated as a convolution of the force gradient *k_ts_* with a semicircular weight function *w*.

In general *k_ts_* is not constant over the oscillation cycle, especially for larger amplitudes *A*. In this case the oscillation of the cantilever has to be taken into account. A derivation of the frequency shift caused by an arbitrary force *F_ts_* is given, for example, in reference [15] based on the Hamilton–Jacobi formalism:

[3]
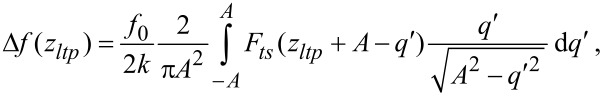


where *z_ltp_* is the lower turnaround point of the oscillation (see Figure 1a). Thus the frequency shift can be calculated by a convolution of the force with an amplitude-dependent weight function. Integration by parts of Equation 3 leads to a more intuitive form:

[4]
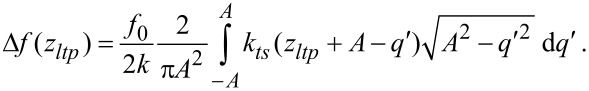


This equation describes the frequency shift Δ*f* as a convolution of the force gradient *k_ts_* with a semicircular weight function with radius *A* (see Figure 1b). Equation 4 is equivalent to Equation 2 upon replacing *k_ts_* with the average force gradient


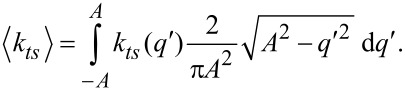


Equation 3 needs to be inverted in order to calculate the force for a given Δ*f*(*z*) curve. Additionally, it enables us to calculate the expected frequency shift for a given force law. In reference [16] analytical functions of Δ*f*(*z*) curves for power and exponential force laws were calculated. A common exponential force law is the force derived from the Morse potential used to describe the bonding between two atoms:

[5]
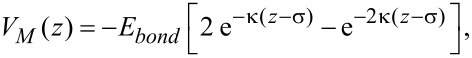


[6]
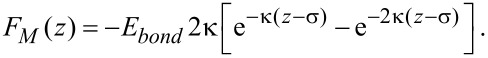


Here *E_bond_* is the bond energy, κ is the decay constant and σ is the equilibrium distance. The frequency shift that is derived from such a Morse force law is given by [16]:

[7]
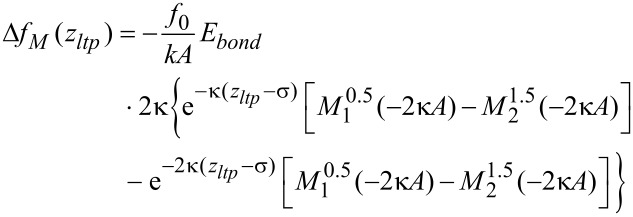


with 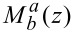 being the Kummer function (see section 13.2.1 in [17]).

Another potential commonly used to describe the interaction between two atoms is the Lennard-Jones potential. In contrast to the Morse potential, the Lennard-Jones potential is based on power functions and has only two parameters, that is, the equilibrium distance σ and the bond energy *E_bond_*:

[8]
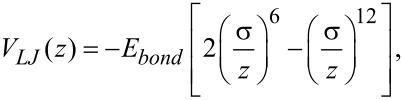


[9]
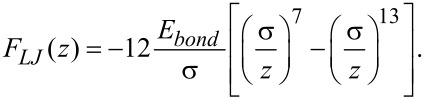


The Lennard-Jones force law leads to the frequency shift [16]:

[10]
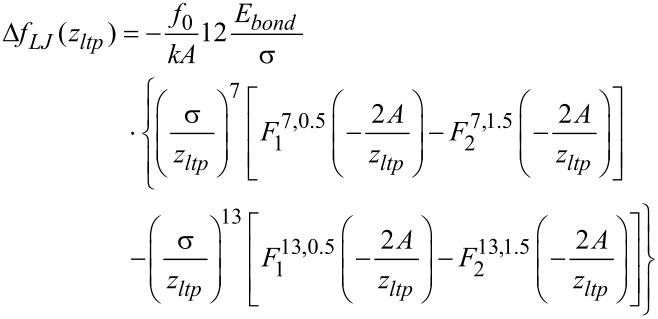


with 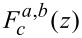 being the hypergeometric function (see section 15.3.1 in [17]). In this work we use both the Morse and the Lennard-Jones force laws as model systems to judge the quality of the force-deconvolution methods.

### Force-deconvolution methods for discrete data

Sader and Jarvis [12] proposed an analytical force-deconvolution method (hereinafter called the Sader–Jarvis method). The force *F_ts_*(*z_ltp_*) is expressed in terms of a Laplace transformation. In doing so, Equation 3 can formally be solved for *F_ts_*. But to calculate the actual expression numerically, part of the Laplace transformed function needs to be approximated by a rational function. Using fractional calculus, Sader and Jarvis provide an equation to recover the force *F_ts_* from a Δ*f*(*z*) in a closed analytical form:

[11]
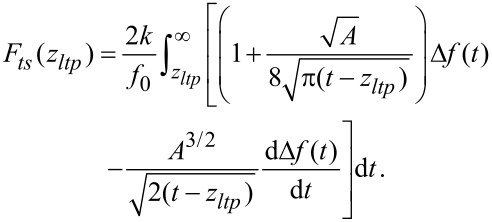


Practically, the frequency shift is not given as an analytical function but in discrete data points Δ*f_i_* = Δ*f*(*z_i_*), *i* = 1,…,*N*. It is convenient to define *z*_1_ as the point of closest approach and *z_i_*_+1_ > *z_i_*, but the data points do not need to be equidistant. Upon implementation of Equation 11, both the derivation and the integration have to be calculated numerically. The derivation is replaced by the difference quotient and the integral is calculated following, for example, the trapezoidal rule:

[12]
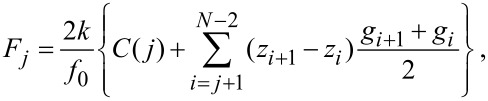


where

[13]
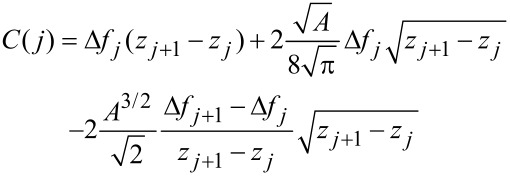


is a correction term. Sader introduced this term in his implementation of the force-deconvolution algorithm [18] to account for the divergence of the integrand in Equation 11 at *t* = *z_ltp_*. The correction term is given by the integration over the interval [*z_j_*,*z_j_* + 1] with Δ*f*(*t*) assumed to be constant. The numerical integration is conducted over the discretized integrand

[14]
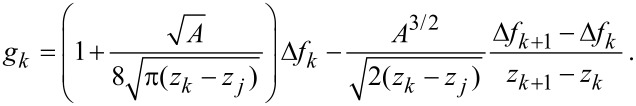


This implementation is of course only one possibility. There are, for example, other algorithms than the trapezoidal rule to perform the numerical integration in Equation 12. Choosing another integration algorithm, the correction term in Equation 13 may become unnecessary (see for example [19]). However, further below we will show that it is not the numerical integration that is the limiting factor in accuracy, but rather the used approximation.

Another method was proposed by Giessibl [13] (hereinafter called the matrix method). This method directly uses the discrete nature of the frequency shift versus distance data. The starting point is also the discretized Equation 3, but the data points Δ*f_i_* = Δ*f*(*z_i_*), *i* = 1,…,*N* must be equidistant: *z_i_* = (*i* − 1)*d* + *z*_1_. Here, *z*_1_ is the first *z* value with nonzero frequency shift coming from far away from the sample. Hence the *z*-axis is opposite to the one used in the Sader–Jarvis method. Equation 3 can be expressed as a matrix equation by appropriate substitution and index shifting:

[15]
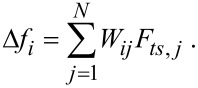


The matrix elements *W_ij_* are given by

[16]
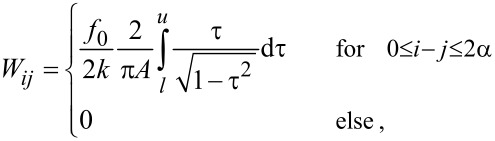


where α = round(*A*/*d*) is the ratio of the amplitude *A* and the step width *d* rounded to the nearest integer. The upper and lower boundaries of the integral are given by

[17]
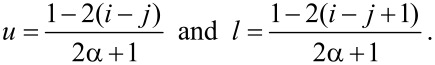


The integral in Equation 16 can be evaluated analytically resulting in 
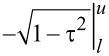
. In order to solve Equation 15 for *F_ts_* the equation needs to be multiplied from the left with the inverse matrix **M** = **W**^−1^ resulting in

[18]
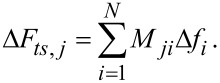


Hence the deconvolution method does not need any approximation and only involves the calculation of the inverse matrix **M**.

It is a common argument that the implementation of the matrix method is more complicated than the Sader–Jarvis method and needs high-performance mathematical software tools [14]. The implementation of Equation 12 and Equation 18 used in this work was done in MATLAB [20], and the scripts are available in Supporting Information File 1. Both implementations are straightforward and work also with the freely available software GNU Octave [21] without modification. As both MATLAB and Octave have built-in optimized routines for matrix operations, the matrix method is slightly faster. This may change upon use of a different implementation or different software.

### Comparison of the force-deconvolution methods

For comparison we consider two theoretical model systems, the Morse potential (Equation 5) and the Lennard-Jones potential (Equation 8). For these model systems we can derive the force laws *F_ts_*(*z*) (Equation 6, Equation 9) and the frequency-shift curves Δ*f*(*z*) (Equation 7, Equation 10) for an FM-AFM force sensor. The calculated frequency-shift curves are deconvoluted back to a force curve *F_S_*_/_*_M_* by using the Sader–Jarvis (S) and the matrix (M) method, respectively.

In order to compare the two deconvolution methods for different force laws, we need a measure for the deconvolution quality. In this work we use the coefficient of determination (CoD)

[19]
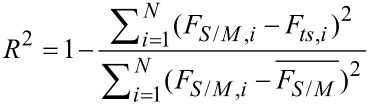


as a measure of the similarity of the modeled force *F_ts_* to the deconvoluted force *F_S_*_/_*_M_*. The 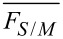 denotes the average of the deconvoluted force and *N* is the number of data points. The CoD is widely used as a measure of the goodness of fit. Generally, 0 < *R*^2^ ≤ 1 holds independently of the number of data points, and the order of magnitude of the force giving a CoD of 1 corresponds to a perfect match. In principle, a negative CoD can also occur, if the force model fits the deconvoluted force worse than just taking the average of the deconvoluted force. As the CoD does not give information about the shape of the deviation, the residuals

[20]
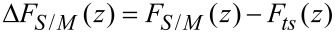


are calculated for selected amplitudes (see below). Both the CoD and the residuals as a measure of the deconvolution quality emphasize the errors at positions with very steep gradients. Therefore, a small shift of the deconvoluted forces, especially in the repulsive regime, leads to strong deviations. However, as the analysis shows, both measures provide a good insight into the deconvolution quality.

Two important parameters of the atomic interaction are the position and the value of the force minimum (maximum attractive force). Therefore, we also compare the deviation from the model values:

[21]
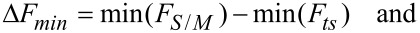


[22]
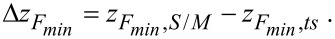


To calculate the frequency shift we chose a tuning fork sensor in the qPlus design [13] with a spring constant of *k* = 1800 N/m and a resonance frequency of *f*_0_ = 32768 Hz. This sensor can operate with very small amplitudes in the picometer range up to large amplitudes in the nanometer range [22]. The amplitude contributes to the deconvolution in a nontrivial way, whereas *k* and *f*_0_ are just linear factors. Therefore, we investigated the amplitude dependence of the deconvolution for the Sader–Jarvis and the matrix method.

We took 500 logarithmically distributed amplitude values *A* in the range from 10 pm to 1 nm. For each amplitude the Morse and Lennard-Jones force and frequency-shift curves were calculated in a *z* range from 0.23 nm to 5 nm with 5000 data points. We assumed an equilibrium distance of σ = 0.235 nm and a bond energy of *E_bond_* = 0.371 aJ, which were previously used to model a silicon–silicon interaction [15]. Additionally, for the Morse potential we assumed a decay constant of κ = 4.25 nm^−1^. This leads to a maximum attractive force of *F_min_* = −790 pN at *z_min_* = 398 pm and *F_min_* = −4.25 nN at *z_min_* = 261 pm for the Morse and Lennard-Jones force laws, respectively.

## Results

### Results for a Morse force law

**Figure 2:**
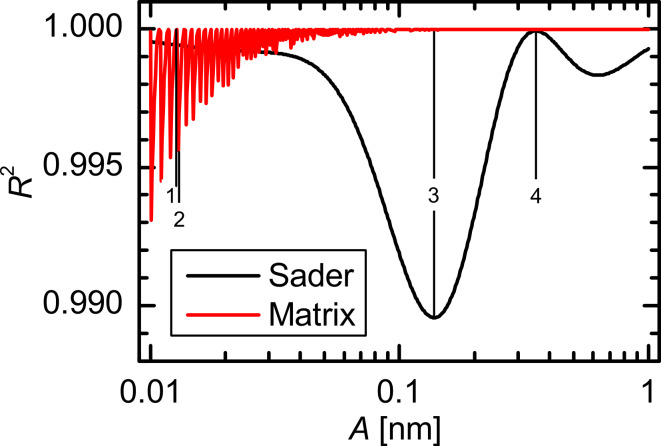
Amplitude dependence of the CoD for the Morse force law. The positions marked with 1, 2, 3, and 4 correspond to the amplitudes 12.8 pm, 12.9 pm, 137 pm and 352 pm, respectively.

Figure 2 shows the amplitude dependence of the CoD *R*^2^ of the Morse force law based on both the Sader–Jarvis and the matrix deconvolution method. Both methods reveal a nontrivial amplitude dependence of the deconvolution quality. Upon using the Sader–Jarvis method the CoD varies continuously reaching the smallest value at an amplitude of *A* = 137 pm and the largest at *A* = 352 pm. With the matrix method the CoD exhibits periodic spikelike features that grow in magnitude as the amplitude is decreased. For larger amplitudes *A* > 100 pm the CoD converges to 1. However, both deconvolution methods have a *R*^2^ > 0.990 over the whole of the considered amplitude range. Thus in terms of the CoD both methods work very well.

**Figure 3:**
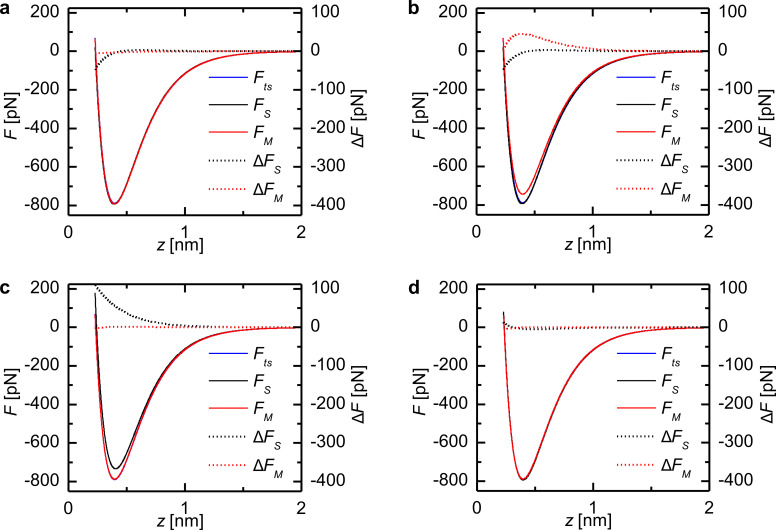
Model force *F_ts_*(*z*), deconvoluted force *F_S_*_/_*_M_*(*z*) and the residuals Δ*F_S_*_/_*_M_*(*z*) for the Morse force law with selected oscillation amplitudes. (a) Amplitude 1 in Figure 2 (12.8 pm) with *R*^2^ ≈ 1 for the matrix method. (b) Amplitude 2 in Figure 2 (12.9 pm) with *R*^2^ = 0.995 for the matrix method. (c) Amplitude 3 in Figure 2 (137 pm) with *R*^2^ = 0.990 for the Sader–Jarvis method. (d) Amplitude 4 in Figure 2 (352 pm) with *R*^2^ ≈ 1 for both methods.

In order to show that these small variations in the CoD represent measurable differences between deconvoluted force and the model force, the model and deconvoluted force curves *F_S_*_/_*_M_*(*z*) and the residuals Δ*F_S_*_/_*_M_*(*z*) are plotted in Figure 3 for selected amplitudes marked in Figure 2. For tip–sample distances greater than 1.5 nm the deviation is below 1 pN. But in the interesting region around the force minimum and in the repulsive regime there are deviations up to 109 pN for both deconvolution methods.

A comparison of the residuals Δ*F_S_*_/_*_M_*(*z*) for an amplitude of 12.8 pm (Figure 3a) and 12.9 pm (Figure 3b) reveals that in case of the matrix method even tiny differences in the oscillation amplitude can have a great effect on the quality of the deconvolution. This manifests as a drop in the CoD from 1 to 0.995. Similarly, strong deviations are present in the residuals for the Sader–Jarvis method. The Sader–Jarvis method leads to a CoD of *R*^2^ = 0.990 at the lowest amplitude of *A* = 137 pm (see Figure 3c) and to *R*^2^ ≈ 1 at the highest amplitude of *A* = 352 pm (see Figure 3d). This rise in the CoD of 0.01 connotes a decrease in the maximum deviation from 109 pN to 13 pN in the residuals. The greatest deviation occurs in the region of the steep gradient to the left of the force minimum, which is caused by a small shift in the *z* values of the deconvoluted force. As can be seen from the force curves, the agreement in that range is still reasonably good.

**Figure 4:**
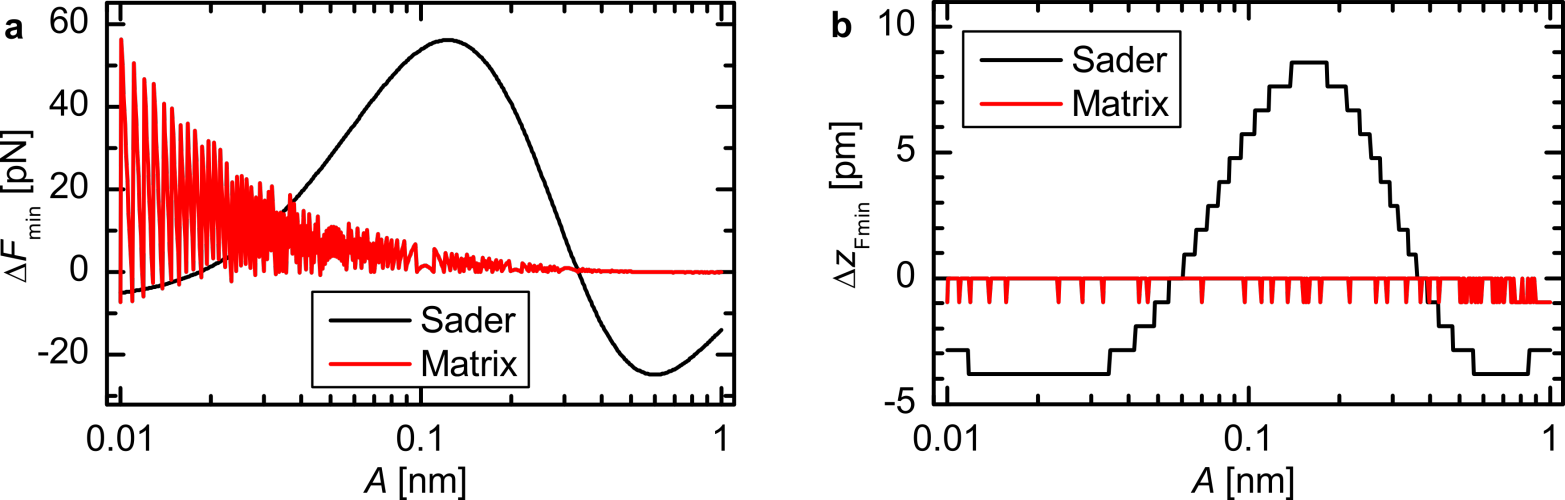
Amplitude dependence of the deviation in magnitude (a) and position (b) from the force minimum for the Morse force law. The steps in (b) are due to the discretization of the *z*-values.

The amplitude dependence of the force minimum Δ*F_min_*(*A*) in Figure 4a has a similar shape to the amplitude dependence of the CoD in Figure 2. The deviations from the force minimum in the Sader–Jarvis method vary continuously, and the largest deviation at an amplitude of *A* = 123 pm almost coincides with the minimum of the CoD at *A* = 137 pm. The matrix method shows spikelike features similar to Figure 2 in the deviation of the force minimum that become greater with decreasing amplitude. However, for amplitudes exceeding 110 pm these deviations become smaller than 3 pN. Whereas the CoD is always above 0.990, the deviations of the force minimum are up to 53 pN corresponding to 7% of the actual value *F_min_* = −790 pN for both deconvolution methods. For most of the considered amplitude range Δ*F_min_* is positive for both methods. Therefore, the absolute value of the deconvoluted maximum attractive force is smaller than the actual maximum attractive force. The deviation in the position can only take an integer multiple of the step width *d* between the *z* values (see Figure 4b). For the Sader–Jarvis method deviations up to nine data points corresponding to Δ*z_Fmin_* = 9 pm occur. The matrix method is in this regard very accurate as there are only deviations of one data point at most.

### Results for a Lennard-Jones force law

**Figure 5:**
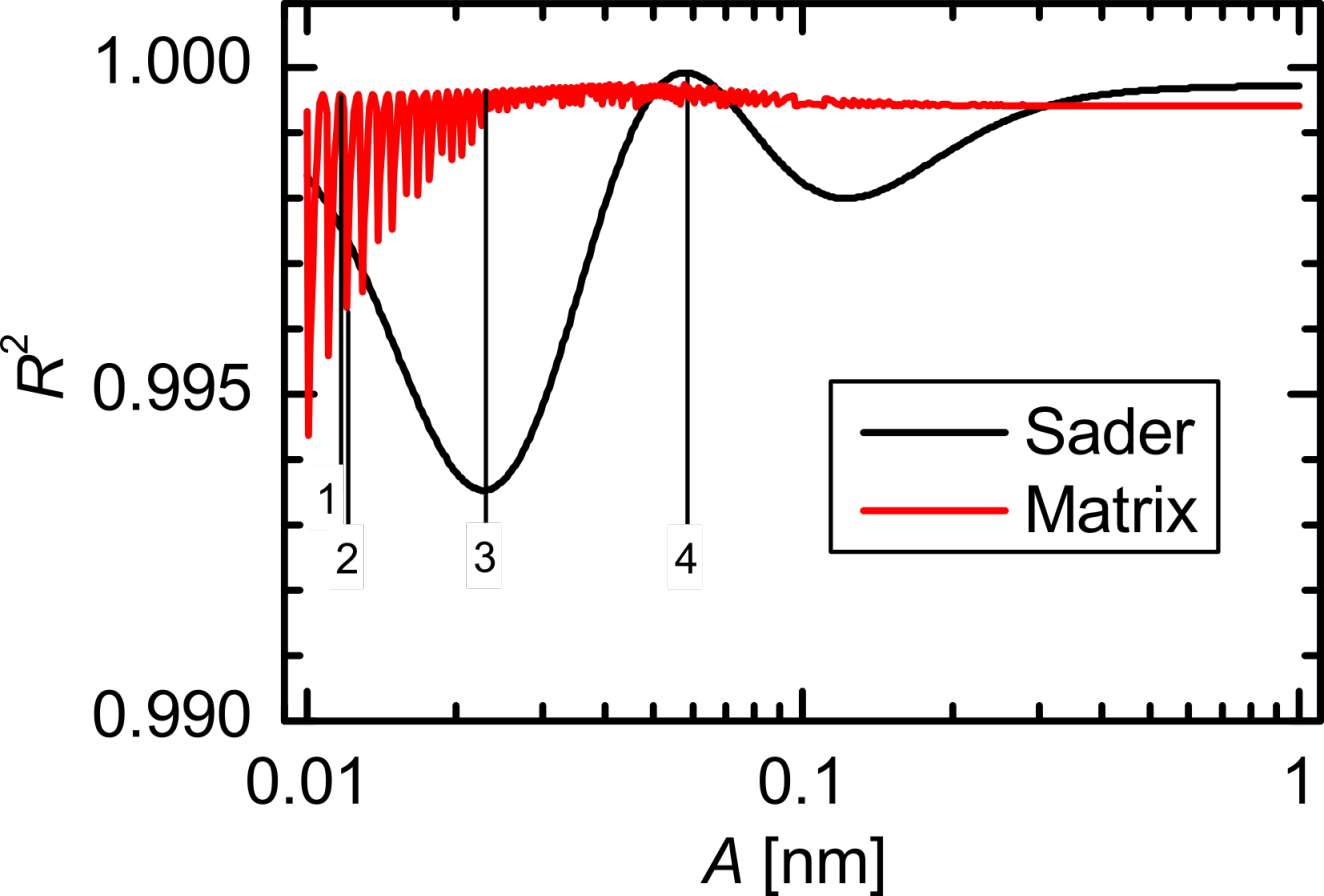
Amplitude dependence of the CoD for a Lennard-Jones force law. The positions marked with 1, 2, 3, and 4 correspond to the amplitudes 11.7 pm, 12.0 pm, 23 pm and 58.3 pm, respectively.

In Figure 5 the amplitude dependence of the CoD for the Lennard-Jones force law is shown. The amplitude dependence is again continuous for the Sader–Jarvis method, but the curve is shifted to smaller amplitudes compared to the Morse force law in Figure 2. The Sader–Jarvis method exhibits minima at amplitudes of 23 pm and 122 pm and a maximum at 58 pm. The matrix method shows again the periodic spikelike features. Additionally, for larger amplitudes the CoD *R*^2^ does not converge to 1. The deconvolution quality expressed by the CoD *R*^2^ ≥ 0.993 is also very high for the Lennard-Jones force law.

**Figure 6:**
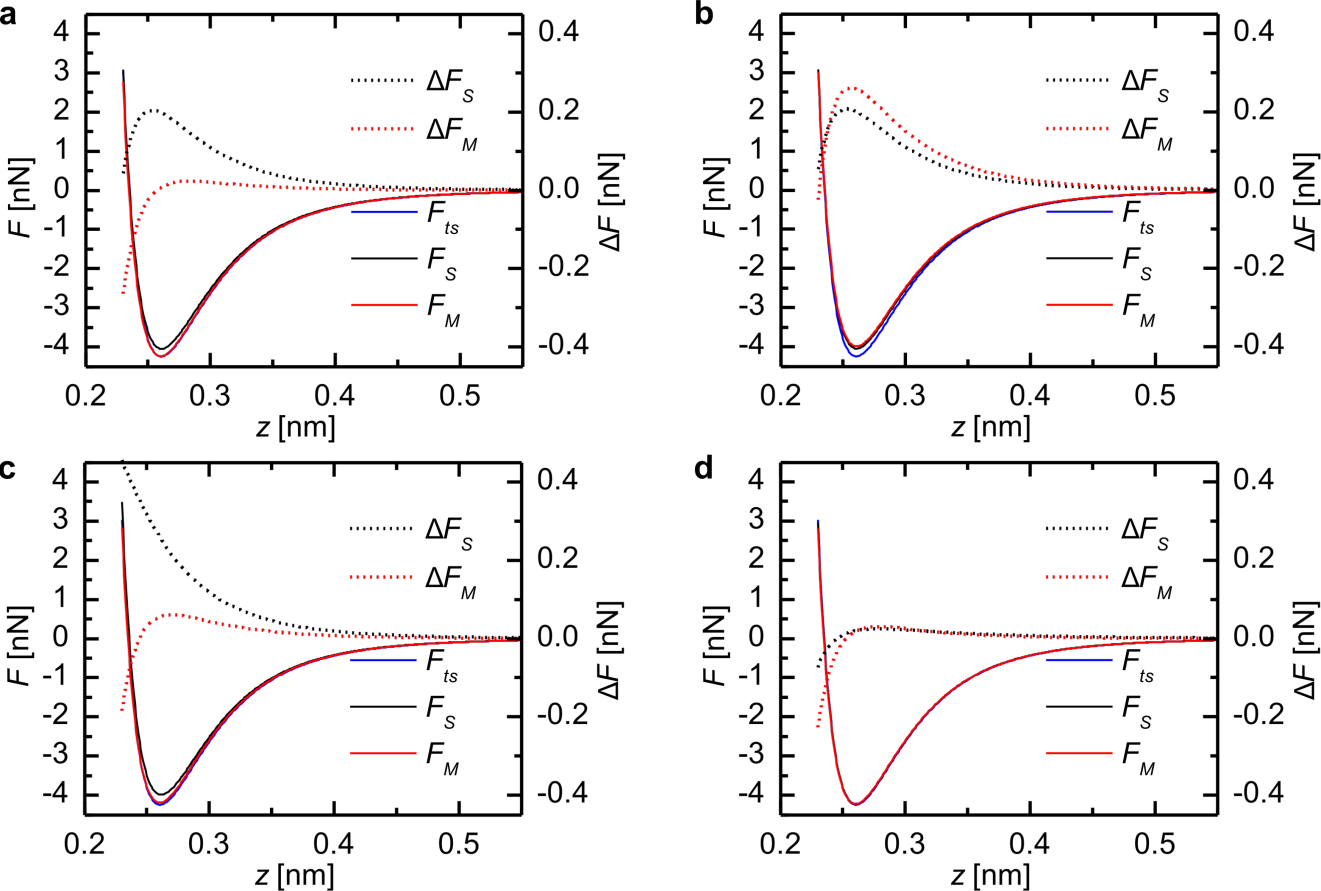
Model force *F_ts_*(*z*), deconvoluted force curves *F_S_*_/_*_M_*(*z*) and the residuals Δ*F_S_*_/_*_M_*(*z*) for the Lennard-Jones force law with selected oscillation amplitude. (a) Amplitude 1 in Figure 5 (11.7 pm) with *R*^2^ = 0.9996 for the matrix method. (b) Amplitude 2 in Figure 5 (12.0 pm) with *R*^2^ = 0.996 for the matrix method. (c) Amplitude 3 in Figure 5 (23 pm) with *R*^2^ = 0.994 for the Sader–Jarvis method. (d) Amplitude 4 in Figure 5 (58.3 pm) with *R*^2^ ≈ 1 for the Sader–Jarvis method.

The deconvoluted force curves and the residuals of the Lennard-Jones force law shown in Figure 6 show significant deviations only for tip–sample distances below 0.55 nm. Comparing the residuals of the matrix method for an amplitude of 11.8 pm (Figure 6a) and 12.0 pm (Figure 6b) also shows a strong deviation of the deconvolution quality due to only a small increase in amplitude, as was observed for the Morse force law. At the first minimum of the CoD for the Sader–Jarvis method the maximum difference between the deconvoluted force and the model force is 460 pN (Figure 6c). For an amplitude of 58.3 pm (Figure 6d) the deviation for the Sader–Jarvis method is only 78 pN corresponding to a CoD of *R*^2^ ≈ 1.

**Figure 7:**
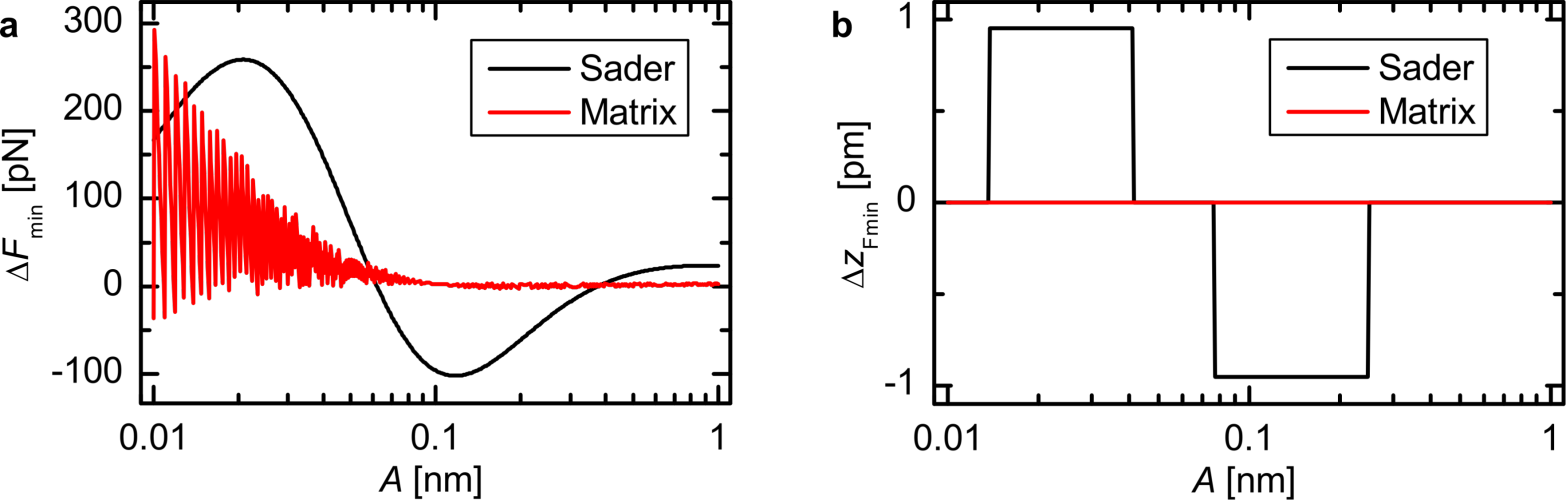
Amplitude dependence of the deviation in magnitude (a) and position (b) from the force minimum for the Lennard-Jones force law. The steps in (b) are due to the discretization of the *z*-values.

For the Lennard-Jones force law the shape of the Δ*F_min_*(*A*) curve (Figure 7a) is similar to the amplitude dependence of the CoD in Figure 5. Using the Sader–Jarvis method the largest deviation appears at an amplitude of 21 pm, approximately where the CoD has its first minimum. At this position, the deconvoluted force minimum is larger than the minimum of the model force. Therefore, the absolute value of the maximum attractive force is smaller than the correct value. At the second minimum of the CoD (*A* = 120 pm) the deviation is negative. For the matrix method most amplitudes result in a positive Δ*F_min_* meaning that the absolute value of the maximum attractive force is underestimated. The deviations from the actual force minimum rise up to 293 pN for the matrix method and up to 259 pN for the Sader–Jarvis method, which is 7% and 6%, respectively, of the correct value *F_min_* = −4.25 nN. The deviations of the position of the force minimum shown in Figure 7b are very small in the case of the Lennard-Jones force law compared to the Morse force law. There are no deviations for the matrix method and the Sader–Jarvis method shows only deviations of one data point at most.

## Discussion

**Figure 8:**
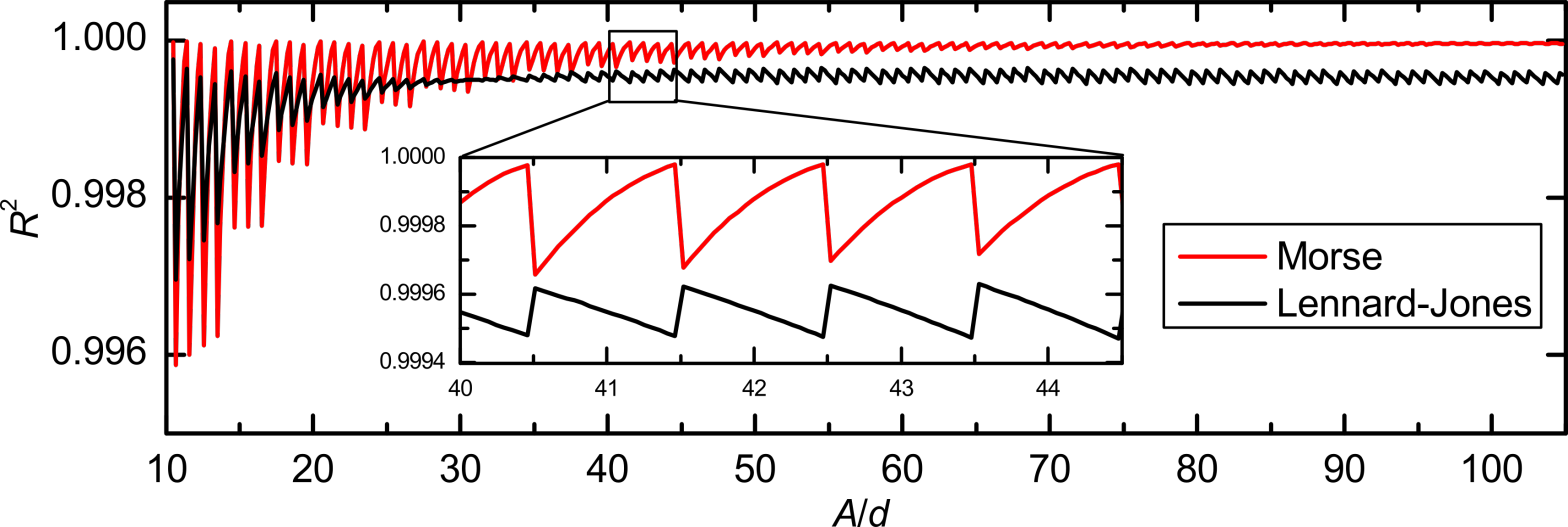
Dependence of the CoD on the ratio *A*/*d* of amplitude and step width for the Morse and the Lennard-Jones force law. The inset shows that the spikes are always at positions *A*/*d* = *n* + 0.5 for both force laws with an integer *n*.

To determine the origin of the amplitude-dependent periodic spikes in the CoD for the matrix method, in Figure 8, we plot the CoD versus the ratio of amplitude and step width *A*/*d* for the Morse and the Lennard-Jones force law. The position of the best deconvolution quality strongly depends on the simulation parameters (force law, amplitude range). But a sharp drop of *R*^2^ for *A*/*d* ≈ *n* + 0.5 is seen for all parameters. Therefore, we suggest using only integer ratios of *A*/*d* as they are furthest away from the singularities.

At first glance the matrix method does not seem to be suitable for small amplitudes. But the drop in the CoD for small ratios *A*/*d* is not related to a shortcoming of the matrix method for small amplitudes but rather to a numerical artifact that is emphasized by using too few data points for the deconvolution. This can be seen as the CoD always goes back to its optimum value even for low ratios of *A*/*d* < 30. If the data points are not given in an appropriate spacing, interpolation methods can be used. This additional data processing increases computational time and memory requirements for the deconvolution. In general it is advisable to use ratios *A*/*d* > 50 as the variation in *R*^2^ becomes very small for greater ratios, whereas a very small ratio *A*/*d* ≤ 1 can even result in a negative CoD.

For the Sader–Jarvis method the situation is different. The *R*^2^(*A*) curves show two distinct minima and one maximum at which the deconvolution quality is optimal. However, the positions of the minima and the maximum are not connected to the ratio *A*/*d*. Therefore, interpolation does not yield a better deconvolution performance.

**Figure 9:**
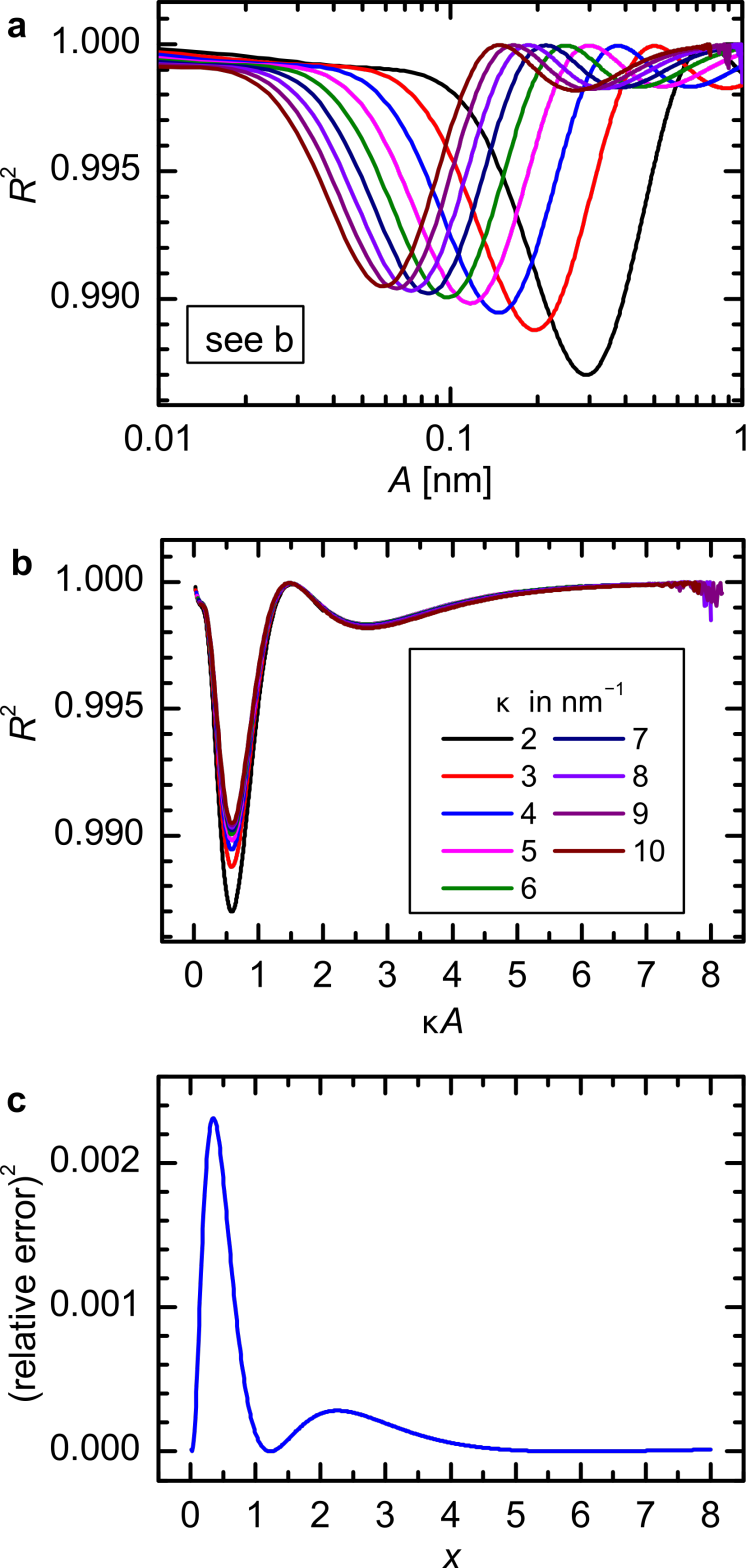
(a) Amplitude dependence of the CoD for Morse force law with different decay constants κ (see legend in (b)). (b) The same data shown in (a) but with a scaled abscissa κ*A*. The minima and maxima coincide on the scaled axis. (c) square of the relative error *SqRE* of the approximation of the function *T*(*x*).

In fact, the deconvolution quality depends on the ratio of the amplitude and the characteristic decay length of the force law. For a Morse force law the decay length is inversely proportional to the parameter κ. In Figure 9a the CoD is shown for Morse force laws with κ’s from 2 nm^−1^ to 10 nm^−1^. We can scale the amplitude axis for every individual CoD curve by κ, as is shown in Figure 9b. The minima and maxima of all curves coincide very well on the scaled axis. In the derivation of Equation 11 the function *T*(*x*) = e^−^*^x^I*_1_(*x*), where *I*_1_(*x*) is the modified Bessel function of the first order [17], is approximated by [12]


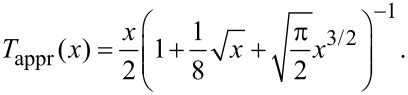


In Figure 9c the squared relative error of this approximation

[23]
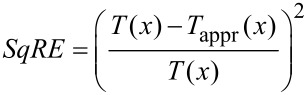


is shown. By comparison of Figure 9b and Figure 9c it is evident that the variation in the deconvolution quality is not a numerical artifact, but an inherent property of the deconvolution method due to this approximation. This approximation exhibits a maximum error of 5%, as already pointed out in [12,23]. This is in concordance with the results presented in this work yielding a maximum error of 7% in the force minimum.

Unfortunately, the optimal and the worst deconvolution lie very close together on the order of the characteristic decay length. For a Morse law the optimal deconvolution is attained for *A* ≈ 1.5 κ^−1^ and the worst for *A* ≈ 0.59 κ^−1^. The deconvolution quality rises again for larger amplitudes *A* > 7 κ^−1^. However, usually amplitudes in the order of the characteristic decay length of the force are desired to obtain the best signal-to-noise ratio [24]. Therefore, in a real experiment it is difficult to judge whether the Sader–Jarvis method will provide an optimal deconvolution.

Besides the deconvolution algorithm, there are other uncertainties in the experimental parameters that have a direct effect on the correctness of the force deconvolution: The stiffness *k*, the amplitude *A* (sensor sensitivity) and the tip–sample distance *z* (*z*-piezo sensitivity). The uncertainties of these parameters are in the range of a few percent. Another important prerequisite to the experimental data is that the frequency shift curves extend far enough from the surface, so that Δ*f*(*z*) and its derivative dΔ*f*(*z*)/dz go to zero, because of the finite number of data points used for the deconvolution.

## Conclusion

We have shown how the deconvolution methods proposed by Sader and Jarvis and Giessibl can be implemented for discrete data points. The analysis of the deconvolution methods has shown that both methods work fine when we are considering the coefficient of determination. However, in certain cases there are significant differences in the deconvolution quality with respect to the amplitude dependence. The deviation from the force minimum was found to be 7% for both methods in the worst case. The matrix method is very sensitive to the ratio *A*/*d* of the amplitude *A* and the step width *d* of the Δ*f*(*z_i_*). The deconvolution can always be optimized by using this method either by taking an integer value of *A*/*d* or by interpolating the data to an integer or very large ratio. The deviations with the Sader–Jarvis method do not originate from the discrete nature of the data points. Therefore, interpolation does not increase the deconvolution quality. The quality is related to the ratio of the amplitude and the characteristic decay length of the force due to the approximation used. For a Morse force law with a decay constant κ it was found that optimal deconvolution is reached for κ*A* = 1.5. Generally, the matrix method provides the higher deconvolution quality, as the data, if necessary, can always be interpolated to equidistant points with a high integer ratio *A*/*d*. If additional data processing is not desired and the data is given in a low or unsuitable ratio *A*/*d*, the Sader–Jarvis method provides a good alternative.

## Supporting Information

File 1 Implementation of the Sader–Jarvis and the matrix force deconvolution algorithm in MATLAB [20].
